# Marked elevation of alkaline phosphatase in an infant treated with levetiracetam: A case report

**DOI:** 10.1016/j.ebr.2026.100896

**Published:** 2026-09-07

**Authors:** Kim Dao, Emmannuella Bonègre, Hassib Chehade, Sébastien Lebon

**Affiliations:** a Service of Clinical Pharmacology, Lausanne University Hospital and University of Lausanne, Lausanne, Switzerland.; b Unit of Paediatric Neurology and Neurorehabilitation, Department Mother-Woman-Child, Lausanne University Hospital, and University of Lausanne, Lausanne, Switzerland.; c Division of Pediatric Nephrology, Department Mother-Woman-Child, Lausanne University Hospital, and University of Lausanne, Lausanne, Switzerland.

**Keywords:** Pediatric, Epilepsy, Levetiracetam, Adverse drug event, Case report

## Abstract

Levetiracetam (LEV) is widely used in the treatment of pediatric epilepsy and is typically well tolerated. We hereby present a case involving an infant who exhibited a notable and isolated elevation of alkaline phosphatase (ALP) during a treatment of LEV, with subsequent normalization following drug cessation. Liver function tests and bone turnover markers remained within normal limits, and the patient was asymptomatic. ALP levels returned to normal within four weeks of discontinuing LEV. LEV may cause significant, reversible ALP elevation in children, most likely due to bone origin. Although rare, this potential adverse effect should be considered, particularly during long-term therapy.

## Highlights

• This case highlights the need for increased awareness of this potential adverse effect, especially in pediatric patients.
• Given the absence of monitoring recommendations for LEV, the question of long-term subclinical effects on bone metabolism is raised.

## Graphical abstract

**Figure:**
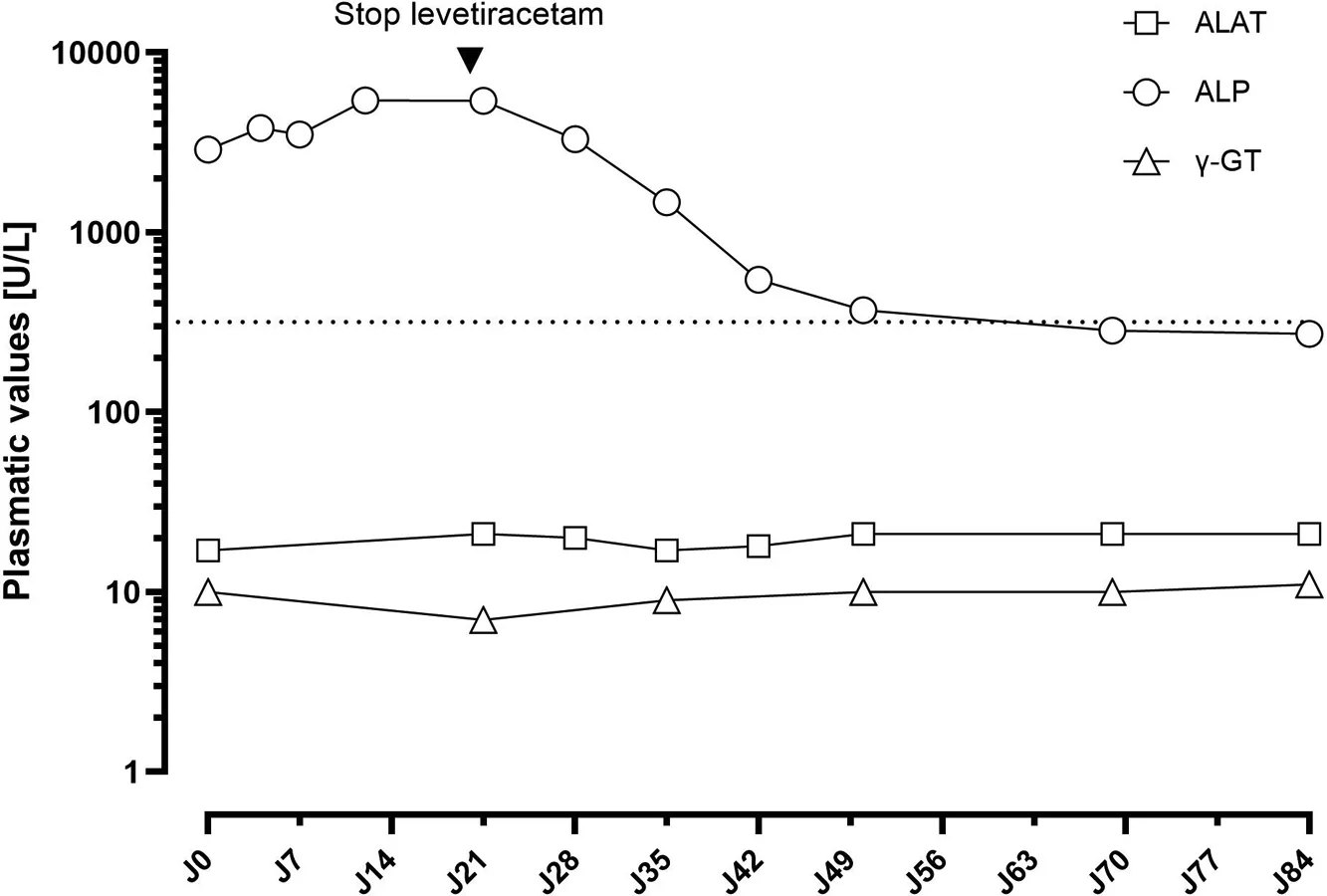

## 1 Introduction

Levetiracetam (LEV) is a structurally unique antiseizure medication (ASM) approved since 1999 for monotherapy in adults with focal seizures and as adjunctive therapy for focal, generalized tonic-clonic, and myoclonic seizures in both adults and children. In Switzerland, LEV is approved for use in term infants over one month of age as adjunctive therapy for focal seizures. [1]

Due to its broad-spectrum efficacy and favorable safety profile, LEV has become one of the most commonly prescribed ASM in children in Western countries. [2], [3] The most frequently reported adverse effects are neuropsychiatric, including somnolence, agitation, and mood disturbances. [1] Liver test abnormalities are uncommon or rare. [1]

The exact mechanism of action of LEV remains unclear. Originally developed as a cognitive enhancer for Alzheimer's disease, LEV is known to interact with multiple neurotransmitter systems, including dopaminergic, cholinergic, and glutamatergic pathways. [4], [5]

LEV has been associated with rare cases of liver enzyme abnormalities, including elevated aminotransferase liver test and, in exceptional cases, liver failure. [6] Isolated increases in alkaline phosphatase (ALP), however, have only been anecdotally reported. To date, only two published case reports describing significant ALP elevations were published, one of which occurred in a pediatric patient. [7], [8] We present a case of a 13-month-old girl who developed a significant, isolated elevation in ALP levels following the initiation of LEV, without clinical or biochemical evidence of liver injury.

## 2 Case presentation

A 13-month-old girl was referred to the pediatric neurology outpatient clinics at the age of 11 months for recurrent prolonged febrile and non-febrile seizures. She exhibited weekly generalized and focal hemiclonic seizures switching from one side to another. Between episodes, her clinical examination and developmental milestones were within normal range. Awake EEG and brain MRI were unremarkable.

Due to the high seizure frequency, oral LEV was introduced initially at 10 mg/kg/day, titrated to 20 mg/kg/day twice daily. Despite good observance, seizure control remained poor and LEV was increased to 30 mg/kg/day but seizures persisted. Early onset epilepsy and seizure semiology (long-lasting hemiclonic seizures with fever sensitivity) were suggestive of a genetic epilepsy related to sodium channelopathy, and valproic acid (VPA) was introduced at 10 mg/kg/day, titrated to 20 mg/kg/day, 4 weeks after LEV initiation.

**Fig. 1:**
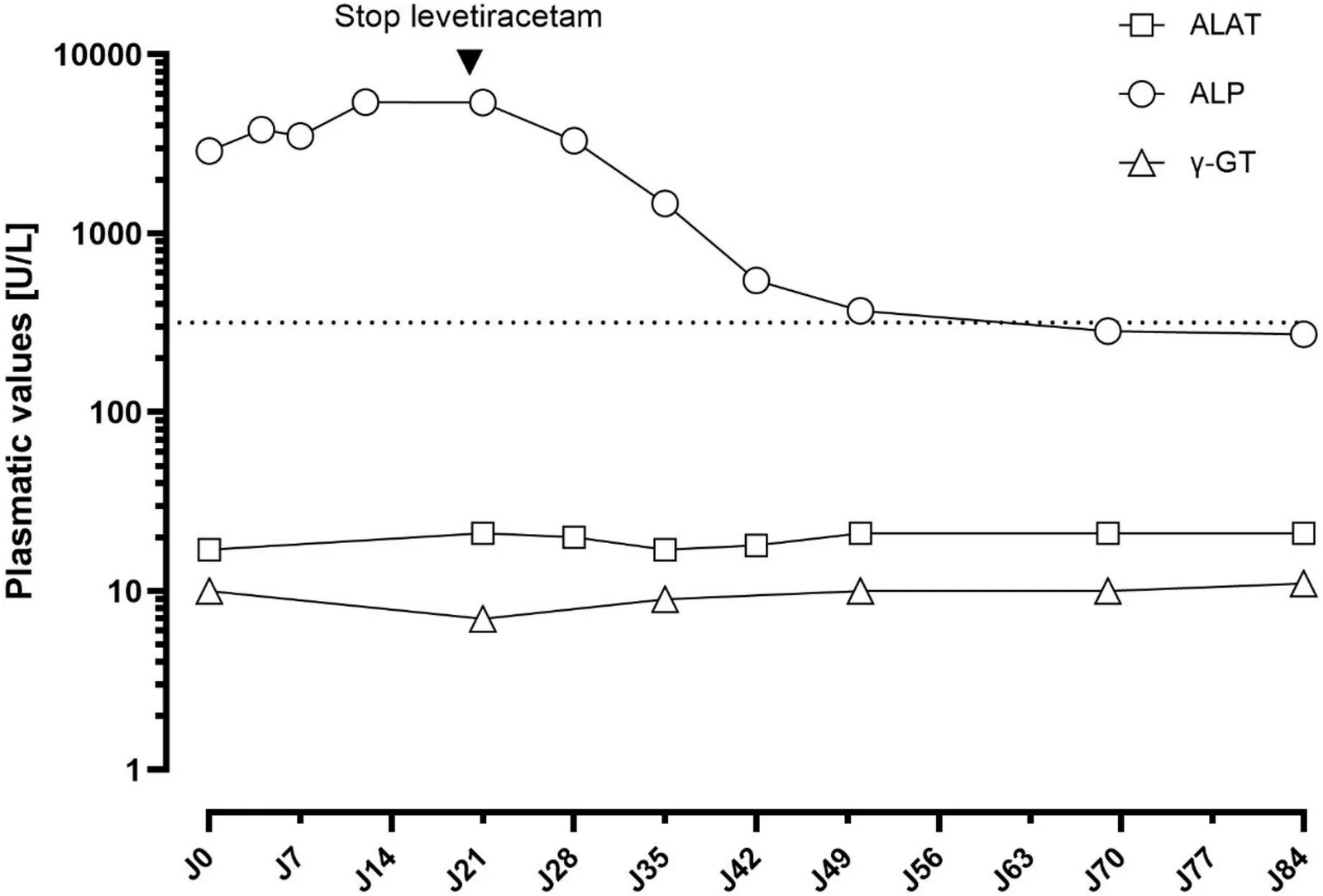
Alkaline phosphatase plasmatic values are represented (open circle) over time, the date levetiracetam treatment was stopped is represented by a downward arrow. A semi-solid line represents the upper normal value for ALP values. Alanine Aminotransferase (ALAT) and Gamma-Glutamyl Transferase are represented over time by open squares and triangles, respectively.

A routine blood test prior to VPA revealed a markedly elevated ALP level of 2894 U/L (reference range for the age 108–317 U/L) while all other parameters remained within normal ranges. Repeated laboratory tests on days 4 and 12 showed a progressive rise in ALP, peaking at 5418 U/L, more than 17 times the upper normal range (Fig. 1).

Despite ongoing seizures, the girl had a normal physical examination, normal growth, without organomegaly, abdominal or limb pain or other discomfort in favor of hepatobiliary dysfunction, intestinal inflammation, malabsorption or bone fracture. Extensive laboratory workup showed normal electrolytes levels (Na 140 mmol/L, total calcium 2.55 mmol/L, corrected calcium 2.47 mmol/L, Mg^2+^ 0.89 mmol/L, phosphate 1.78 mmol/L), normal hepatic enzymes level (ALT 34 U/L, AST 17 U/L, gamma-GT 10 U/L, total bilirubin 3 μmol/L, direct bilirubin <2 μmol/L), normal 25-OH vitamin D level, parathormone (22.4 ng/L) and blood cell count. Creatine kinase and lactate dehydrogenase levels were respectively moderately and slightly increased (max level 318 U/L (reference range 25–140 U/L) and 431 U/L (reference range 155–345 U/L), respectively). In absence of clear explaining factors of this elevated serum ALP, a potential role of LEV therapy was considered and discontinued. ALP serum decreased to 1472 U/L 3 weeks later. Thirty days after the LEV discontinuation, ALP level returned to normal (369 U/L), CK values remained elevated at 301 U/L. No measure of bone ALP were available.

A genetic epilepsy panel revealed a heterozygous *de novo SCN8A* c.647 T > C class IV variant, confirming a diagnosis of SCN8A-related developmental and epileptic encephalopathy. At follow-up five months later, seizures had become shorter but occurred weekly under a new regimen of topiramate and clobazam. ALP remained within normal range, while CK levels stayed mildly elevated. The patient's neurodevelopment plateaued, consistent with the underlying genetic diagnosis.

This case of adverse drug reaction was anonymously reported to Swissmedic according to Federal Act on Therapeutic Products in Switzerland.

## 3 Discussion

This case demonstrates a rare, isolated and significant asymptomatic elevation in ALP following the introduction and dose escalation of LEV in an infant with a monogenic developmental and epileptic encephalopathy. The first measure of elevated ALP was detected one month after LEV initiation during a routine check-up – where LEV was the only medication administered. The absence of alternative medical explanation, the temporal association, the progressive decline in ALP following LEV dechallenge, despite the introduction of VPA, and the description of a similar case in the literature all support a causal relationship, further substantiated by a Naranjo Adverse Drug Reaction Probability Score indicating a “probable” adverse reaction. [7] Rechallenge with LEV would have strengthened the causal hypothesis but was considered unethical in this patient given the suspected causal link and parental concerns. The introduction of VPA during LEV dechallenge represents a confounding factor, as VPA is known to affect bone metabolism. However, if VPA were contributory, one would expect a rise in ALP rather than the observed normalization.

In addition, we found two reports of ALP elevation probably linked to LEV. A 10-month-old girl with posttraumatic epilepsy, who had been treated for 5 months with LEV (250 mg/day, *i.e.* 27.8 mg/kg/day) presented with a significant but asymptomatic elevation of ALP values, peaking at 4557 U/L and accompanied by a mild increase in CK-MB levels (50 U/L, 0–24 U/L), lactate dehydrogenase (320 U/L, 109–245 U/L) and bone ALP (> 300 U/L, 0–200 U/L). Pre-LEV ALP values were normal (162 U/L). Abdominal CT and long bones X-ray were normal. LEV was discontinued, and the course was rapidly favorable, with a decrease in ALP values in 20 days. [7] Elevated liver enzymes (ALP, 567 IU/L along with elevated alanine aminotransferase, 289 IU/L; aspartate aminotransferase 184 IU/L) were reported in a 62-year-old female treated with levetiracetam 500 mg twice daily for seizure prophylaxis after 10 days of treatment. Enzymes returned to baseline after levetiracetam was discontinued. [8]

While several ASMs such as VPA or carbamazepine are known to cause liver injury [9], [10] and therefore require regular monitoring, biochemical abnormalities associated with LEV are rarely reported, their true frequency remains unknown, and no guidelines currently recommend routine blood monitoring. [1] In our case, the ALP elevation suggested a bone-related rather than a hepatic origin. This hypothesis was supported by normal hepatic enzyme levels, the absence of historical or clinical signs of bone injury and the existence of a similar case report. Bone origin remains a diagnosis of exclusion, as no bone-specific ALP fraction was measured to support this hypothesis. Intestinal ALP isoenzymes elevation could also be a differential diagnosis though there were no clinical indicators.

The mild elevation of CK was attributed to frequent convulsive seizures rather than LEV toxicity. There were no clinical signs suggestive of myopathy. Eventually, the muscle origin of elevated ALP appears unlikely considering normalization of ALP after LEV discontinuation and persistent mild CK elevation, and the discrepancy between the CK elevation (2× the upper normal range) and the markedly elevated ALP (>10× the upper normal range).

Interestingly, osteoporosis and multiple fractures were reported in patients with *SCN8A* developmental and epileptic encephalopathy. [11], [12] In animal models, *SCN8A* inactivation led to reduced trabecular number and thickness, cortical bone thinning, and increased bone resorption markers. A role for the Nav 1.6 channel in excessive osteoclastogenesis and increased bone resorption is hypothesized, though secondary muscle atrophy and immobilization may represent the primary driver. [11] Whether the LEV-associated ALP elevation reflects mutation-specific susceptibility, an idiosyncratic LEV adverse effect, or both remains to be determined.

We performed an analysis of data gathered up to November 2025 in VigiBase Database (the WHO global database of individual case safety reports (ICSRs)) [13] which included 75 cases of increased blood ALP associated with levetiracetam (LEV). The disproportionality analysis did not identify a safety signal for this association (IC 0.1, IC025–0.3). Out of these 75 cases, only 20 cases concerned isolated elevated blood ALP without reported concomitant liver injury where LEV was a suspected drug. In this analysis, considering the 20 cases with isolated elevated blood ALP, the median time to onset was 12 days after LEV was introduced, with a range going from 1 day to 296 days, although chronology was often missing (*n* = 12). LEV was the only suspected agent in 17 cases (85%). In these cases, the causality assessment for LEV was rated as *possible* for 8 cases, as *probable* in 1 case and unspecified for the others. Median age was 20 years (interquartile range 13–54 years old), including 7 pediatric cases, age was missing in 3 cases. A predominance of female gender is observed (*n* = 15, 75%). Reactions were reported as recovered or recovering in 25% of the ICRS, not recovered in 30%, and was otherwise unknown. In cases where elevated blood ALP was reported as recovered/recovering (*n* = 5), LEV was stopped in only 2 cases (including the present case report). Details of these cases were unavailable, as is often the case with spontaneous reports in pharmacovigilance databases. Temporal relationship, female sex, and absence of alternative suspected agents are all consistent with the reported case. Data gathered within VigiBase comes from a variety of sources, and the probability that the suspected adverse effect is drug-related is not the same in all cases. Of note, it does not represent the opinion of the Uppsala Monitoring Centre or the World Health Organisation.

The impact of epilepsy and ASM on bone metabolism is increasingly recognized. Older enzyme-inducing ASM have been frequently associated with a reduction in serum calcium, phosphate and an increased serum ALP, likely due to an enhanced conversion of vitamin D to its inactive moiety. [14], [15], [16] Although LEV is not a classically enzyme-inducing drug, some studies have noted mild effects on bone markers. A prospective study in 26 epileptic patients (aged 1–15 years) reported slight but significant ALP increases at 2 and 6 months after LEV initiation, without an increase in γ-GT, suggesting a bone origin. [17] In a cross-sectional adult study, long term LEV use has been associated with decreased bone mineral density and increased markers of bone resorption, such as procollagen type 1 N-terminal propeptide. However, other bone metabolism indicators did not change significantly with levetiracetam, including levels of 1,25-dihydroxyvitamin-D. [18] A recent meta-analysis, including pediatric and adult patients, confirmed an association between LEV and decreased serum calcium, recommending routine monitoring of bone metabolism-related indicators. [19]

## 4 Conclusion

Although LEV is widely considered as a safe and effective ASM, it might cause a significant, isolated, and reversible elevation in serum ALP, potentially of bone origin. The underlying mechanism remains unclear. This case highlights the need for increased awareness of this potential adverse effect, especially in pediatric patients. Given the absence of monitoring recommendations for LEV, this raises the question of whether some children may experience long-term subclinical effects on bone metabolism. Further prospective studies are needed to better understand and assess the prevalence and implications of ALP elevations in LEV-treated patients.

## CRediT authorship contribution statement

**Kim Dao:** Writing – review & editing, Writing – original draft. **Emmannuella Bonègre:** Writing – original draft, Investigation, Data curation. **Hassib Chehade:** Writing – review & editing, Investigation. **Sébastien Lebon:** Writing – review & editing, Writing – original draft, Visualization, Validation, Supervision, Methodology, Investigation, Formal analysis, Data curation, Conceptualization.

## Declaration of competing interest

The authors declare that they have no known competing financial interests or personal relationships that could have appeared to influence the work reported in this paper.
